# Association of Selenium Levels with Gestational Diabetes Mellitus: An Updated Systematic Review and Meta-Analysis

**DOI:** 10.3390/nu14193941

**Published:** 2022-09-23

**Authors:** Hamdan Z. Hamdan, Sumaia Zaki Hamdan, Ishag Adam

**Affiliations:** 1 Department of Basic Medical Sciences, Unaizah College of Medicine and Medical Sciences, Qassim University, Unaizah 56219, Saudi Arabia; 2 Faculty of Medicine, Al-Neelain University, Khartoum 12702, Sudan; hamdanzh@neelain.edu.sd; 3 Department of Obstetrics and Gynecology, Unaizah College of Medicine and Medical Sciences, Qassim University, Unaizah 56219, Saudi Arabia; ia.ahmed@qu.edu.sa

**Keywords:** gestational diabetes mellitus, hyperglycemia, meta-analysis, pregnancy, selenium, trace elements, systematic review

## Abstract

Several studies have investigated the association between selenium levels and gestational diabetes mellitus (GDM); however, their results are not conclusive. This systematic review and meta-analysis aimed to update and draw conclusions regarding the evidence from published studies that investigated selenium levels in relation to GDM. PubMed, Google Scholar, Cochrane Library and ScienceDirect were searched for studies related to selenium and GDM, published from the inception of each database through to July 2022. The meta-analysis was conducted by measuring the standardized mean difference (SMD) between the selenium levels of women with GDM and those pregnant without GDM (control group). Stratified meta-analysis, meta-regression analysis and reporting bias were applied. The “meta” package in the open-access software R was used to analyze all of the data. A total of 12 studies, including 940 pregnant women with GDM and 1749 controls met this study’s inclusion criteria. The selenium levels were significantly lower in women with GDM compared with the control group (SMD = −0.66; 95% confidence interval (CI): (−1.04, −0.28); *p* ≤ 0.001). Due to significant heterogeneity (*I*^2^ = 94%, Cochrane Q = 186.7; *p* ≤ 0.0001), the random-effects model was followed. The stratified meta-analysis showed that the selenium levels were lower in the cases compared with the normal controls in the third trimester (SMD = −1.85 (−3.03, −0.66); *p* ≤ 0.01). The same trend was observed in the studies published before the year 2014 (SMD = −0.99 (−1.70, −0.28); *p* ≤0.01) and those published in or after 2014 (SMD = −0.45 (−0.90, 0.00); *p* = 0.05). None of the investigated covariates in the meta-regression analysis (each study’s geographic location, trimester of selenium quantification, World Bank economic classification, method of selenium determination, study design, study quality score, publication year and study’s sample size) were significantly associated with the selenium SMD. The current evidence indicates that selenium levels are lower among women with GDM in comparison to those without GDM; however, after the correction of the reporting bias, the result was no longer significant. Further studies with more prospective designs are needed to confirm this evidence and explain the function of selenium in GDM throughout pregnancy.

## 1. Introduction

Gestational diabetes mellitus (GDM) is a common endocrine problem during pregnancy, with a prevalence of 15% of all pregnancies worldwide [1,2]. GDM is defined as the onset of glucose intolerance for the first time during the pregnancy of previously healthy women [1]. GDM can lead to macrosomia, which can complicate the delivery process [3]. Moreover, GDM can result in the development of type 2 diabetes mellitus (T2DM) later in a woman’s life, as well as preeclampsia in subsequent pregnancies. Likewise, the offspring of mothers with GDM are at higher risk of developing diabetes mellitus (DM) and cardiovascular diseases than the offspring of women without GDM. This higher risk stems from the epigenetic changes that occur in the growing fetus during intrauterine life [4].

The definite cause of GDM has not yet been fully explored. However, many risk factors that may contribute to the susceptibility to GDM, such as maternal obesity, past history of GDM and family history of DM [5,6,7,8], have been identified. Oxidative stress has been postulated to explain the relations between these observed risk factors and GDM [9]. According to this hypothesis, the degree of insulin resistance is correlated with accumulating free radicals, and inversely correlated with anti-oxidant levels [10]. Oxidative stress causes direct damage to the β-cells of the pancreas and, as a corollary, increases insulin resistance, which in turn manifests as hyperglycemia [11].

Selenium is an important trace element necessary for optimal several physiological processes [12]. Selenium acts as an active site component of the anti-oxidant enzyme glutathione peroxidase [13]. This intra-cellular enzyme deactivates free radicals and helps decrease the level of oxidative stress inside the cells [13]. Moreover, an experimental study demonstrated that selenium as a metal can bind to and activate insulin receptors and shows insulin-mimicking activity [14], such as lowering blood glucose levels, increasing glucose uptake by tissues, and enhancing the cellular utilization of glucose [15]. Furthermore, supplemental selenium intake by GDM patients resulted in improved control of blood glucose and reduced oxidative stress levels in [16].

Several studies reported an association between selenium deficiency and T2DM [17,18,19]. Additionally, several reports associated selenium with GDM [20,21,22,23,24,25,26]. However, these studies had contradictory results. While some studies observed an association between low selenium levels and the development of GDM [20,21,22,23], others reported no association [24,25], and one reported an association between GDM and a high level of selenium [26].

Two previous systematic reviews and meta-analyses published in 2015 and 2016 reported significantly lower levels of selenium among cases with GDM compared with normal controls [27,28]. However, some recently published studies had inconclusive results [29,30,31]. Therefore, this study was conducted to re-assess and update the current knowledge about the association between selenium levels and GDM. The findings of this systematic review and meta-analysis could serve as valuable evidence to guide interventions, such as clinical trials, or even preventive measures, such as early diagnosis.

## 2. Materials and Methods

### 2.1. Study Protocol

The protocol for this systematic review and meta-analysis was listed in an international database of prospectively registered systematic reviews in health and social care (PROSPERO), with the registration number CRD42021239431 [32].

### 2.2. Search Strategy

The Preferred Reporting Items for Systematic Reviews and Meta-Analyses (PRISMA) guidelines were strictly followed [33]. An electronic literature search for all reports that assessed the association between selenium levels and GDM, published at any point from database inception through to 10 July 2022, was conducted in PubMed, Cochrane Library, Google Scholar and ScienceDirect using Boolean connectors (AND, OR, NOT) in conjunction with MeSH and non-MeSH terms in the appropriate search space (Table S1). The following search strategies were used, which were prepared according to the Population Intervention Comparison Outcome Study (PICOS) design protocol:

P (population): pregnancy OR pregnant women OR gestation;

I (intervention): selenium OR selenate OR Se;

C (comparison): euglycemic OR normal pregnancy OR healthy pregnancy;

O (outcome): gestational diabetes OR gestational diabetes mellitus OR gestational hyperglycemia OR GDM;

S (study type): case–control OR cross-sectional OR cohort.

Two investigators (SZH and IA) independently screened and carefully chose eligible studies for this meta-analysis after perusal, reading and assessment. Any disagreement was resolved by a discussion with the referee investigator (HZH). The search was aided by LitSuggest [34], an automated literature searching and prioritizing tool. All results excluded by LitSuggest were double-checked by the reviewers before final exclusion.

### 2.3. Inclusion Criteria

The articles were included if the studies (1) investigated the association between selenium levels and GDM; (2) employed case–control, cohort or cross-sectional designs; (3) reported mean (standard deviation (SD)), median (interquartile) or median (range) values of the selenium levels in the case and the control groups and provided a measurement unit; (4) described the methods used to measure the selenium levels and measured the selenium levels in blood, plasma or serum and (5) were written in the English language.

### 2.4. Exclusion Criteria

Systematic review articles, case reports, commentaries, editorials, clinical trials, abstracts and duplicate publications were excluded. The studies that reported selenium levels from hair samples and those written in languages other than English were also excluded.

### 2.5. Primary Outcome Definition

The primary outcome of this study is the investigation of the association between selenium levels and the development of GDM in comparison to healthy normoglycemic pregnant women. According to Carpenter and Coustan [35], GDM is briefly defined as the presence of 2 values above the following thresholds: fasting plasma glucose > 95 mg/Dl (or 1 h post-load glucose > 180 mg/dL, 2 h post-load glucose > 155 mg/dL and 3 h post-load glucose > 140 mg/dL).

### 2.6. Assessment of Risk of Bias

Two investigators (IA and SZH) assessed the quality of the included studies by using the Newcastle–Ottawa Scale (NOS) for the case–control and cohort studies; the modified version of the NOS was used to assess the cross-sectional studies [36]. Three main principles were assessed: participant selection, comparability of study groups and ascertainment of outcomes of interest in each study. The maximum NOS score is nine stars. The studies that earned ≥7 stars were considered high-quality studies. Any possible risk of bias in each included study was also assessed and depicted using Cochrane Collaboration’s tool.

### 2.7. Data Extraction

The Joanna Briggs Institute Meta-Analysis of Statistics Assessment and Review Instrument was used to extract the data from each eligible study [37]. Briefly, for each eligible study, the following data were extracted: first author name, publication date, country of study, study design, numbers of participants in the case and control groups, the levels of selenium and the methods used to determine them, maternal age, gestational age at time of selenium sample collection and pre-gestational body mass index (BMI). When the studies reported selenium levels using median (interquartile) values [25,38], the investigators re-calculated the average (SD) using a previous equation [39].

### 2.8. Statistical Analyses

The open-source statistical software R 4.0.3 (The R Foundation for Statistical Computing, Vienna, Austria) was used to measure the standardized mean differences (SMDs) in selenium levels between the case and control groups by applying the function “metacont” in the *meta* package [40]. The effect size (SMD) was calculated following Hedges’ g method, by subtracting the mean of the selenium levels in the case group from the mean in the control group, which was then standardized by the pooled change in the SD [41]. The heterogeneity of the included studies was assessed using Cochrane Q and *I^2^*. Cochrane Q with *p* < 0.010 and *I^2^* > 50% indicated evidence of inter-study heterogeneity [42].

Due to the high inter-study heterogeneity, the random-effects model was followed in this meta-analysis. Sensitivity analysis was applied to recognize any study that significantly changed the selenium SMD upon exclusion. A graphical funnel plot and a quantitative Egger’s test were used to investigate reporting bias. Any evidence of reporting bias was further examined by means of the trim-and-fill method in order to correct the asymmetry in the funnel plot. Stratification meta-analysis was conducted by grouping the studies according to the trimester in which the blood samples were collected for selenium measurement (first, second and third trimesters) and the study’s year of publication (before 2014 and in or after 2014), as we noticed that studies published after 2014 had smaller selenium SMDs than those published before 2014. Meta-regression analysis was performed to assess the relations between the selenium SMD and the following factors: study quality score, publication year, study continent, selenium measurement trimester, selenium quantification methods, regional economic ranking and study sample size. A *p*-value < 0.05 was considered statistically significant in this study.

## 3. Results

### 3.1. Studies Selection

**Figure 1:**
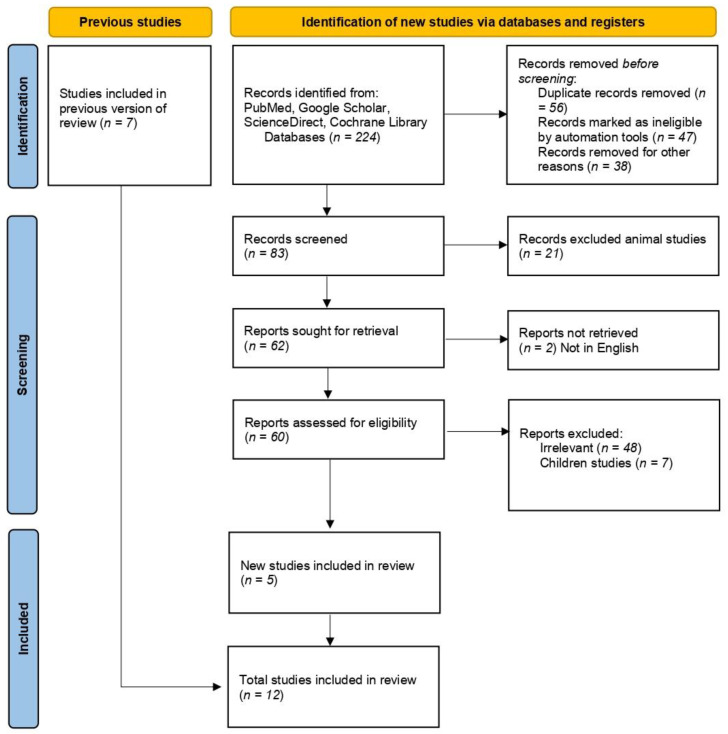
Flow diagram of the search process.

The preliminary search in the databases retrieved a total of 224 articles. Out of this total, 56 were excluded as duplicates and 85 were irrelevant. The two investigators screened the titles and the abstracts of the remaining 83 articles. After screening, 21 were removed as they were animal studies, and 2 more were removed because they not written in English. Out of the 60 articles that were retrieved, 48 were found to be irrelevant, and 7 were primarily about children. Finally, 12 studies were included for meta-analysis, as shown in Figure 1 [20,21,22,23,24,25,26,29,31,38,43,44].

### 3.2. Characteristics of the Included Studies

In this systematic review and meta-analysis, the included 12 studies comprised a total of 940 GDM cases and 1749 controls [20,21,22,23,24,25,26,29,31,38,43,44]. The number of GDM cases in the included studies ranged from a low of 10 [24] to a high of 305 [38], while the numbers of participants in the control groups ranged from a low of 11 [24] to a high of 453 [31].

Of the 12 studies, 8 were case–control [20,22,24,25,26,38,43,44], 3 were cohort [23,29,31], and only 1 was cross-sectional [21]. Six studies were performed on the Asian continent (three in China, two in Kuwait and one in Iran) [20,22,24,29,38,43]. Five studies were conducted in Europe (two in Turkey and one each in Italy, Hungary and Poland) [21,23,26,31,44], and only one study was conducted in Africa (Sudan) [25] (Table 1). Four studies involved women in the second trimester [21,23,26,44], four had participants in the third trimester [22,24,25,43], three had participants in the first trimester [29,31,38], and one study sampled women in both the second and third trimesters [20]. Six studies [20,21,29,38,43,44] were conducted in middle-income countries, five [22,23,24,26,31] in high-income countries and only one [25] in a low-income country.

Six studies were published before 2014 [20,21,22,23,24,26], and another six were published in or after 2014 [25,29,31,38,43,44]. Seven studies [21,22,23,24,25,26,43] used atomic absorption spectroscopy (AAS), four [29,31,38,44] used inductively coupled plasma mass spectrometry (ICPMS), and one [20] used atomic fluorescence spectroscopy (AFS). All the included studies were considered to be of high quality (Table S2). Figure S1 shows the results of the study assessment for the risk of bias using the Cochrane tool.

### 3.3. Overall Meta-Analysis

**Figure 2:**
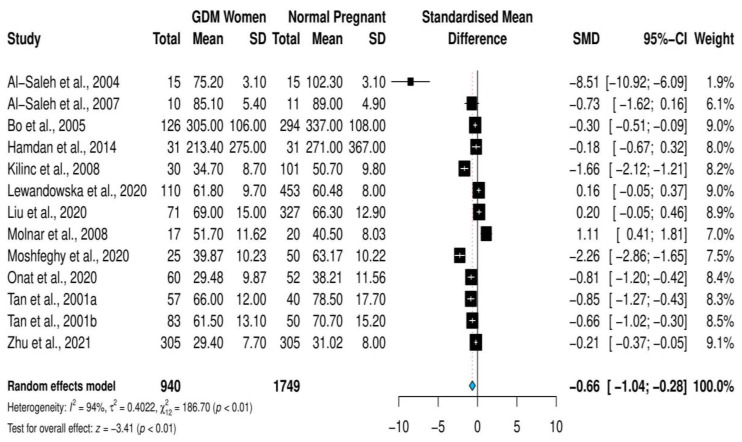
Forest plot of the association between selenium SMD levels and gestational diabetes mellitus [20,21,22,23,24,25,26,29,31,38,39,43,44].

The overall pooled estimate of the standardized mean difference of selenium levels was SMD = −0.66; 95% CI, (−1.04, −0.28); *p* = 0.0007 (Figure 2). The measures of heterogeneity were significant (*I*^2^ = 94%, Cochrane Q = 186.7; *p* ≤ 0.001). Accordingly, the random-effects model was followed.

Sensitivity analysis revealed that none of the included studies significantly changed the SMD of selenium upon deletion, as shown in Figure S2 (see also Figure S3). Therefore, the use of the random-effects model was continued, without excluding any studies.

### 3.4. Stratified Meta-Analysis and Meta-Regression

**Figure 3:**
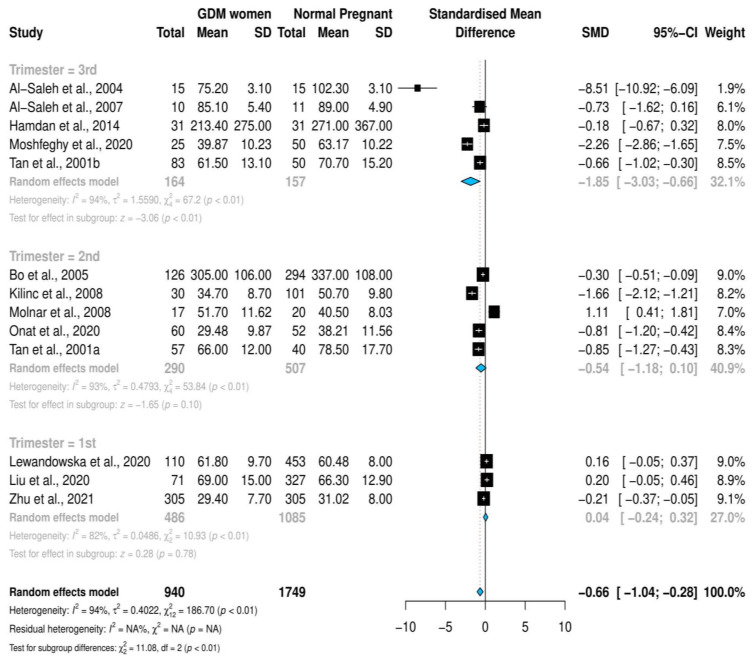
Forest plot of the association between selenium SMD levels and gestational diabetes mellitus; stratified meta-analysis according to trimester at which selenium was measured [20,21,22,23,24,25,26,29,31,38,43,44].

**Figure 4:**
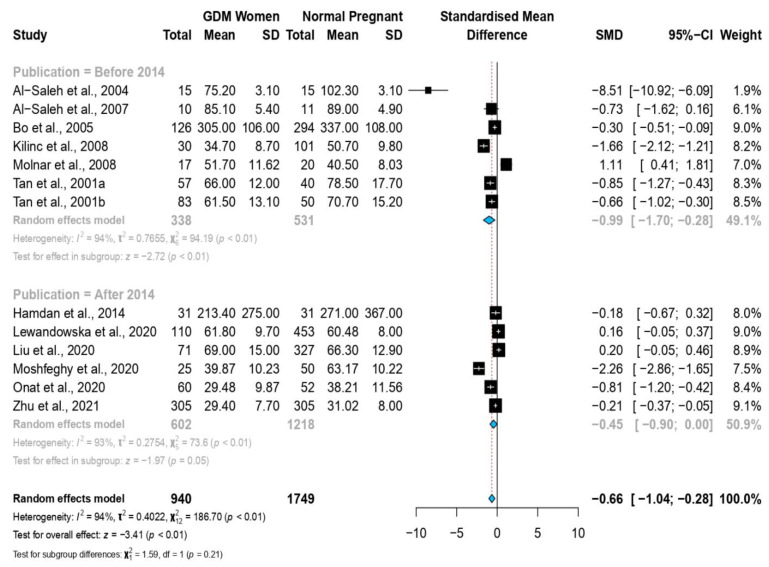
Forest plot of the association between selenium SMD levels and gestational diabetes mellitus; stratified meta-analysis according to year of publication [20,21,22,23,24,25,26,29,31,38,43,44].

Stratified meta-analysis based on the trimester of selenium quantification, revealed that the heterogeneity level increased with the trimester. Likewise, the SMD of selenium levels increased along with the trimester, and was the highest during the third trimester (first trimester: SMD = 0.04 (−0.24, 0.32); second trimester: SMD = −0.54 (−1.18, 0.10); third trimester: SMD = −1.85 (−3.03, −0.66); *p* ≤ 0.01); see Figure 3. Moreover, the grouping of the studies according to the year of publication revealed that the SMD of the selenium levels in the articles published before 2014 (SMD = −0.99 (−1.70, −0.28); *p* ≤ 0.01) was higher than in the studies published in or after 2014 (SMD = −0.45 (−0.90, 0.00); *p* = 0.05); see Figure 4.

In the meta-regression analysis, none of the investigated factors—geographic location, trimester of selenium quantification, World Bank economic ranking, methods of selenium quantification, study design, study quality score, publication year and study sample size—showed any evidence of a relation with the overall estimate (Table 2).

### 3.5. Reporting Bias Assessment

A graphical funnel plot was generated to show any asymmetry in the plotted studies that might indicate the presence of reporting bias. A careful examination of the plot showed an asymmetry in the pattern of the depicted studies (Figure S3). Quantitively, Egger’s test showed evidence of reporting bias (*t* = −2.34; *p* = 0.032). Therefore, the trim-and-fill method was applied, which corrected the plot asymmetry by adding four studies. The newly corrected pooled measures for the 16 studies were SMD = −0.15; 95% CI: (−0.57, 0.26); *p* = 0.473 (Figure S4).

## 4. Discussion

The growing incidence of GDM, which affects approximately one in ten pregnant women [45], draws both scientists’ and clinicians’ attention to the investigation of the possible causes of GDM. Insulin resistance with elevated levels of oxidative stress was reported among pregnant women with GDM in [46]. Among its different functions in the body’s cells, selenium acts against free radicals and reduces oxidative stress, which is believed to improve insulin resistance [10,11]. Many observational studies in different countries noted lower selenium levels in pregnant women with GDM compared with euglycemic pregnant women [20,22,23,43,44]. However, some controversial findings have also been reported [24,25,26,29]. These observations led Asemi et al. (2015) to conduct a clinical trial that provided 200 µg of selenium supplement to women with GDM for six weeks [16]. The study reported three outcomes: first, improved glycemic control; second, reduced oxidative stress; and third, amelioration of inflammatory markers. More recently, Najib et al. (2020) conducted a trial that administered to women with GDM half of the selenium dose used in the study of Asemi et al. for 12 weeks, and reported no effect on glucose homeostasis [47]. Taken together, these premises may provide the rational link between low selenium levels and the development of GDM.

The major finding of this systematic review and meta-analysis was that lower selenium levels were found in women with GDM than in women with no GDM. This finding is in line with the results of two previous systematic reviews and meta-analyses [27,28]. However, the present meta-analysis investigated 12 studies involving 940 GDM cases compared with the 6 studies involving 147 patients in the meta-analysis by Askari et al. (2015) and the 7 studies involving 569 patients in the meta-analysis by Kong et al. (2016) [27,28].

Moreover, this study included studies that employed a cohort design, which investigated selenium levels during the first trimester and followed up on the women throughout their pregnancies [23,29,31]. In contrast, the meta-analyses by Askari et al. (2015) and Kong et al. (2016) included only case–control and cross-sectional designs [27,28]. These cohort studies provide more insights about the causality of and opportunity for predicting GDM from the first trimester [23,29,31].

Although this study also had significant heterogeneity, as did the two previous meta-analyses, this study relied on more in-depth stratified meta-analysis and sensitivity analysis as well as reporting-bias tracing, which resulted in more significant findings. One of the findings in the current study is that selenium levels significantly decreased as the gestational age advanced. For example, the maximum decrement in selenium was observed in the third trimester. This result is in accordance with Kong’s (2016) meta-analysis [27]. This reduction in selenium could be due to physiological hemodilution and escalating maternal and fetal needs [48,49]. Moreover, as pregnancy advanced, the levels of oxidative stress and lipid peroxidation also increased, which reduced the selenium levels [50]. The present study found that selenium levels were higher in GDM cases than in the control groups in the studies published both before and after 2014. However, the studies published after 2014 showed a smaller SMD than in earlier studies. This can be attributed to the trimester in which the selenium levels were quantified, as all studies published before 2014 measured selenium levels during the third or the second trimester, while 50% of the studies published after 2014 measured selenium levels during the first trimester. The methods of selenium quantification should also be kept in mind. As 66% of the studies published after 2014 used ICPMS, while AAS was used in 83% of the studies published before 2014, it is worth mentioning that ICPMS has a low detection limit for measuring trace elements, including selenium, compared with AAS [51]. Notably, measurements of trace elements in recent years show a trend toward the use of ICPMS over AAS, which is considered a relatively old technology.

In a previous meta-analysis [27], it was mentioned that selenium levels were significantly affected by the study’s geographic location. This is explained by the variations in the selenium contents of the diets of the different populations under study. Dietary habits during pregnancy may also determine the amount of selenium that can be consumed by pregnant women. The present study did not apply stratified meta-analysis based on geographic location because of the risk of bias; otherwise, the findings would have been different [27].

Despite the subgroup analysis, the level of heterogeneity did not decrease significantly. Therefore, sensitivity analysis was performed in an attempt to identify the study or studies that could significantly change the overall effect and had heterogeneity. However, none of the investigated studies was found to significantly change the overall effect. This study found evidence of reporting bias, and four studies were estimated to be possibly missed in this meta-analysis. The supposed four missed studies influenced the overall effect and shifted it from a significant to an insignificant level. Although in their meta-analysis Kong et al. observed an asymmetrical distribution of studies in the funnel plot, no evidence of reporting bias was found, which was also the case in the meta-analysis by Askari et al. [27,28]. The observed reporting bias in the present study can be partially attributed to the study selection criteria—specifically the inclusion of only articles written in English. A future meta-analysis may be enhanced by adding studies published in languages other than English.

Although this study updates the current literature, it has some limitations that must be acknowledged when interpreting the findings. First, reporting bias was corrected by adding four studies. However, other types of bias, such as selection bias, could not be avoided. Second, although cohort studies were included, which could explain the causality, the conclusions of these studies were neither uniform nor clear enough to formulate a conclusion about causality. Third, BMI and participant age were not reported in all the included studies. Consequently, a more in-depth analysis regarding these two important covariates could not be conducted. Fourth, the included studies reported varying levels of selenium while providing minimal or no details about validation or quality control for the method of determination. Fifth, the diagnostic criteria for GDM were neither uniform nor reported in all of the studies. This could alter the diagnostic threshold for glucose levels, and hence the diagnoses.

## 5. Conclusions

This updated systematic review and meta-analysis indicates that selenium is lower among women with GDM in comparison to pregnant women with no GDM, who comprised the control group. However, the difference was not statistically significant after the correction of the reporting bias by means of the trim-and-fill method. There is a need for further research that includes more well-designed cohort studies, as well as the measurement of selenium levels throughout the trimesters, to more clearly explain the kinetics of selenium in GDM.

## Figures and Tables

**Table 1:** Features of studies investigating selenium levels included in the overall meta-analysis.

Study, Year (Study Type)	Study Location	Diagnostic Criteria	Selenium Measurement Methods	GDM Group Sample Size Selenium Level Mean (SD) µg/L		Control Group Sample Size Selenium Level Mean (SD) µg/L	
Al-Saleh et al., 2004 [22] (Case–control)	Kuwait	NA	AAS	15	75.2 (3.1)	15	102.3 (3.1)
Al-Saleh et al., 2007 [24] (Case–control)	Kuwait	NA	AAS	10	85.1 (5.4)	11	89 (4.9)
Bo et al., 2005 [23] (Cohort)	Italy	Carpenter and Coustan	AAS	126	305 (106)	294	337 (108)
Hamdan et al., 2014 [25] (Case–control)	Sudan	Carpenter and Coustan	AAS	31	213.4 (275)	31	271 (367)
Kilinc et al., 2008 [21] (Cross-sectional)	Turkey	Carpenter and Coustan	AAS	30	34.7 (8.7)	101	50.7 (9.8)
Lewandowska et al., 2020 [31] (Cohort)	Poland	IADAPSG	ICPMS	110	61.8 (9.7)	453	60.48 (8)
Liu et al., 2020 [29] (Cohort)	China	IADAPSG	ICPMS	71	69 (15)	327	66.3 (12.9)
Molnar et al., 2008 [26] (Case–control)	Hungary	WHO	AAS	17	51.7 (11.62)	20	40.5 (8.03)
Moshfeghy et al., 2020 [43] (Case–control)	Iran	Carpenter and Coustan	AAS	25	39.87 (10.23)	50	63.17 (10.22)
Onat et al., 2020 [44] (Case–control)	Turkey	Carpenter and Coustan	ICPMS	60	29.48 (9.87)	52	38.21 (11.56)
Tan et al., 2001a [20] (Case–control)	China	NA	AFS	57	66 (12)	40	78.5 (17.7)
Tan et al., 2001b [20] (Case–control)	China	NA	AFS	83	61.5 (13.1)	50	70.7 (15.2)
Zhu et al., 2021 [38] (Case–control)	China	IADAPSG	ICPMS	305	29.4 (7.7)	305	31.02 (8)

AAS, atomic absorption spectroscopy; AFS, atomic fluorescence spectroscopy; IADAPSG, International Association of Diabetes and Pregnancy Study Groups; ICPMS, inductively coupled plasma mass spectrometry; NA, not available; WHO, World Health Organization.

**Table 2:** Meta-regression analysis of the factors possibly affecting selenium SMD.

Covariate	Coefficient	95% Confidence Interval	Standard Error	*p*-Value
**Continent** Non-Europe	−0.848	(−5.213, 3.516)	2.227	0.703
**Trimester of Selenium Measurement** 2nd Trimester 3rd Trimester	−2.871 −3.283	(−10.294, 4.552) (−10.173, 3.606)	3.787 3.515	0.448 0.350
**Economic Classification** Middle and Low Income	−0.162	(−3.510, 3.186)	1.708	0.924
**Study Design** Case–Control Studies	1.467	(−2.691, 5.624)	2.121	0.489
**Selenium Detection Method** AFS ICPMS	3.058 −0.771	(−1.850, 7.968) (−6.406, 4.864)	2.504 2.875	0.222 0.788
**Year of Publication**	−0.046	(−0.514, 0.421)	0.845	0.845
**Study NOS Quality Score**	1.794	(−0.565, 4.154)	0.136	0.136
**Sample Size**	−0.003	(−0.013, 0.007)	0.555	0.555

## Data Availability

The datasets used and/or analyzed during the current study are available within the paper.

## Supplementary Materials

The following supporting information can be downloaded at: https://www.mdpi.com/2072-6643/14/19/3941/s1, Table S1: Searching strategies for PubMed, Cochrane library, Google Scholar and ScienceDirect. Table S2: The Modified Newcastle-Ottawa Scale rating for included studies: (*,** OR *** means criteria fulfilled/maximum score = 9). Table S3: Sensitivity analysis of the meta-analysis. Figure S1: Cochrane collaboration tool for risk of bias assessment. Figure S2: Sensitivity analysis. Figure S3: Funnel plot of the reporting bias. Figure S4: Trim-and-fill graph.
