# Supplementary material for: Association of Selenium Levels with Gestational Diabetes Mellitus: An Updated Systematic Review and Meta-Analysis
Source: Nutrients. 2022 Sep 23;14(19):3941. doi: 10.3390/nu14193941 (PMC9570773; doi:10.3390/nu14193941)
Supplement: Supplementary file 1 [file nutrients-14-03941-s001.zip › nutrients-1851131-supplementary.pdf]

Table. S1 Searching strategies for PubMed, Cochrane library, Google Scholar and ScienceDirect

**PubMed**	**Search strategy**
#1	Pregnant women [MeSH]
#2	Gestation [MeSH]
#3	#1 OR #2
#4	Selenium [MeSH]
#5	Selenate
#6	Se
#7	#4 OR #5 OR #6
#8	Pregnancy-Induced Diabetes [MeSH]
#9	Gestational Diabetes [MeSH]
#10	Gestational Diabetes Mellitus [MeSH]
#11	Gestational hyperglycaemia
#12	GDM
#13	#8 OR #9 OR #10 OR #11 OR #12
#14	Normal pregnancy
#15	Healthy pregnant
#16	Normoglycemic
#17	#14 OR #15 OR #16
#18	#3 AND #7 AND #13 AND #17
**Cochrane library**	**Search strategy**
#1	Pregnancy-Induced Diabetes [ti, ab, kw]
#2	Gestational Diabetes [ti, ab, kw]
#3	Gestational Diabetes Mellitus [ti, ab, kw]
#4	Gestational hyperglycaemia [ti, ab, kw]
#5	GDM [ti, ab, kw]
#6	#1 OR #2 OR #3 OR #4 OR #5
#7	Pregnant women [ti, ab, kw]
#8	Gestation [ti, ab, kw]
#9	#7 OR #8
#10	Selenium [ti, ab, kw]
#11	Selenate [ti, ab, kw]
#12	Se [ti, ab, kw]
#13	#10 OR #11 OR #12
#14	Normal pregnant [ti, ab, kw]
#15	Healthy pregnant[ti, ab, kw]
#16	Normoglycemic [ti, ab, kw]
#17	#14 OR #15 OR #16
#18	#6 AND #9 AND #13 AND #17
**Google Scholar**	
#1	allintitle: selenium "Gestational Diabetes Mellitus" OR "GDM" OR "Gestational hyperglycaemia" OR "Pregnancy-Induced Diabetes"
**ScienceDirect**	
#1	(("Gestational Diabetes Mellitus" OR "GDM" OR "Gestational hyperglycaemia" OR "Pregnancy-Induced Diabetes") AND "selenium")

Table S2. The Modified Newcastle-Ottawa Scale rating for included studies: (*, OR *** means criteria fulfilled/maximum score =9)**

**Studies**	**Selection**	**Comparability**	**Outcome**	**Total number of stars**
Al-Saleh *et al.,* 2004 [22]	**	***	**	7
Al-Saleh *et al.,* 2007 [24]	**	***	**	7
Bo *et al.,* 2005 [23]	***	***	**	8
Hamdan *et al.,* 2014 [25]	****	***	**	9
Kilinc *et al.,* 2008 [21]	**	***	**	7
Lewandowska *et al.,* 2020 [31]	***	***	**	8
Liu *et al.,* 2020 [29]	***	***	**	8
Molnar *et al.,* 2008 [26]	****	***	**	9
Moshfeghy *et al.,* 2020 [41]	****	***	**	9
Onat *et al.,* 2020 [42]	****	***	**	9
Tan *et al.,* 2001*a* [20]	**	***	**	7
Tan *et al.,* 2001*b* [20]	**	***	**	7
Zhu *et al.,* 2021 [38]	****	***	**	9

Table S3. Sensitivity analysis of the meta-analysis

Study omitted	SMD	95% CI	*P*-value	I^2
**None**	**-0.657**	**[-1.03; -0.279]**	**<0.0001**	**93.6%**
Al-Saleh *et al.,* 2004 [22]	-0.499	[-0.836; -0.162]	0.003	92.3%
Al-Saleh *et al.,* 2007 [24]	-0.654	[-1.047; -0.262]	0.001	94.1%
Bo *et al.,* 2005 [23]	-0.722	[-1.161; -0.284]	0.001	94.1%
Hamdan *et al.,* 2014 [25]	-0.705	[-1.107; -0.303]	<0.001	94.1%
Kilinc *et al.,* 2008 [21]	-0.547	[-0.912; -0.181]	0.003	92.7%
Lewandowska *et al.,* 2020 [31]	-0.751	[-1.166; -0.336]	<0.001	93.4%
Liu *et al.,* 2020 [29]	-0.749	[-1.157; -0.341]	<0.001	93.6%
Molnar *et al.,* 2008 [26]	-0.782	[-1.163; -0.401]	<0.001	93.6%
Moshfeghy *et al.,* 2020 [41]	-0.503	[-0.858; -0.148]	0.005	92.4%
Onat *et al.,* 2020 [42]	-0.647	[-1.048; -0.246]	0.001	93.9%
Tan *et al.,* 2001*a* [20]	-0.643	[-1.042; -0.244]	0.001	93.9%
Tan *et al.,* 2001*b* [20]	-0.665	[-1.071; -0.259]	0.001	94.0%
Zhu *et al.,* 2021 [38]	-0.746	[-1.206; -0.286]	0.001	94.1%

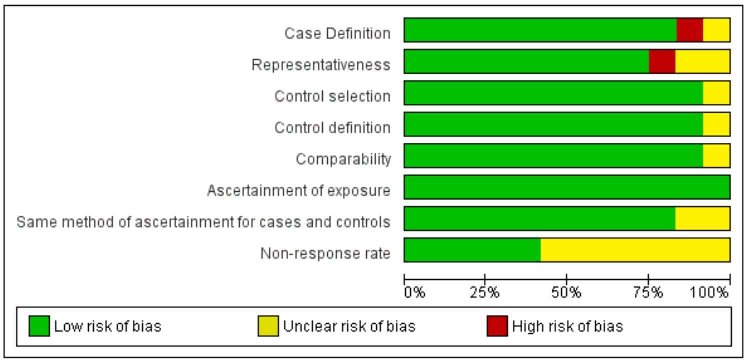

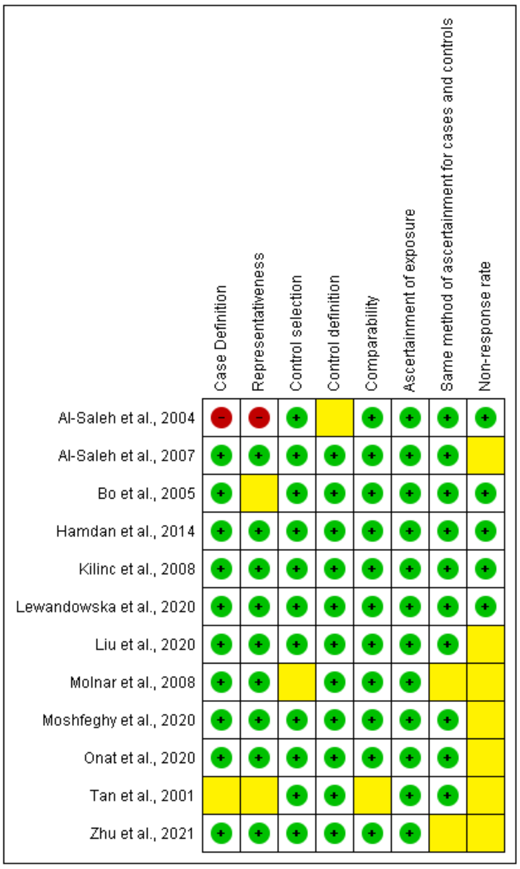

Figure S1: Cochrane collaboration tool for risk of bias assessment[20–26,29,31,38,43–44].

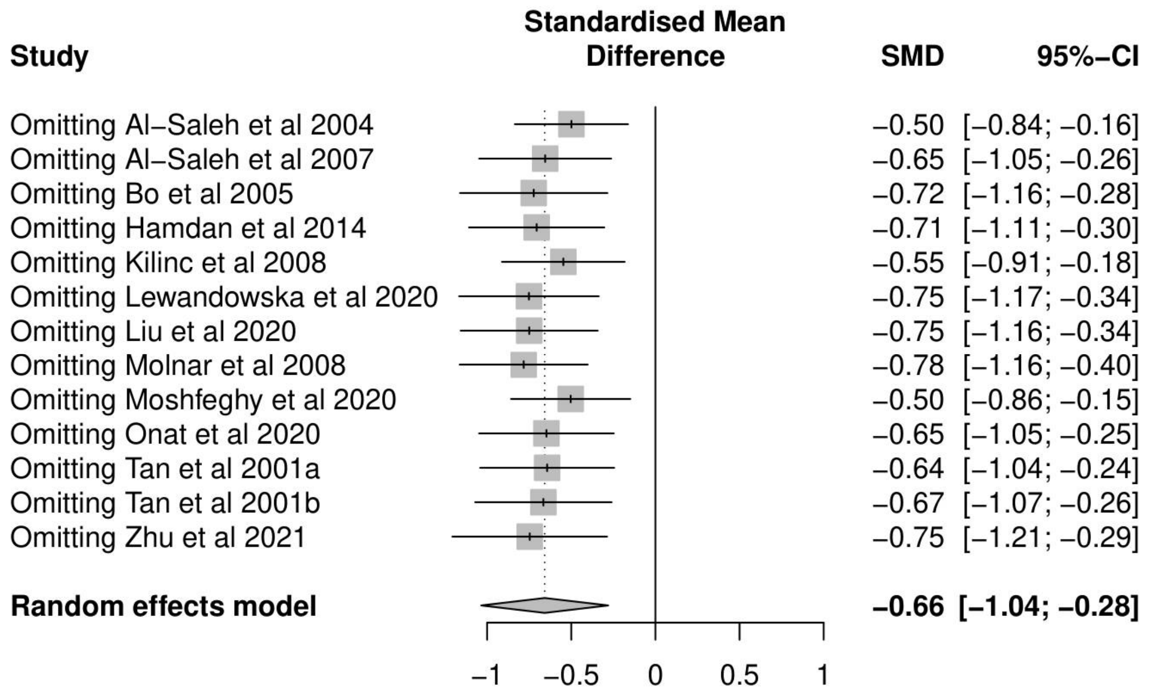

Figure S2: Sensitivity analysis[20–26,29,31,38,43–44].

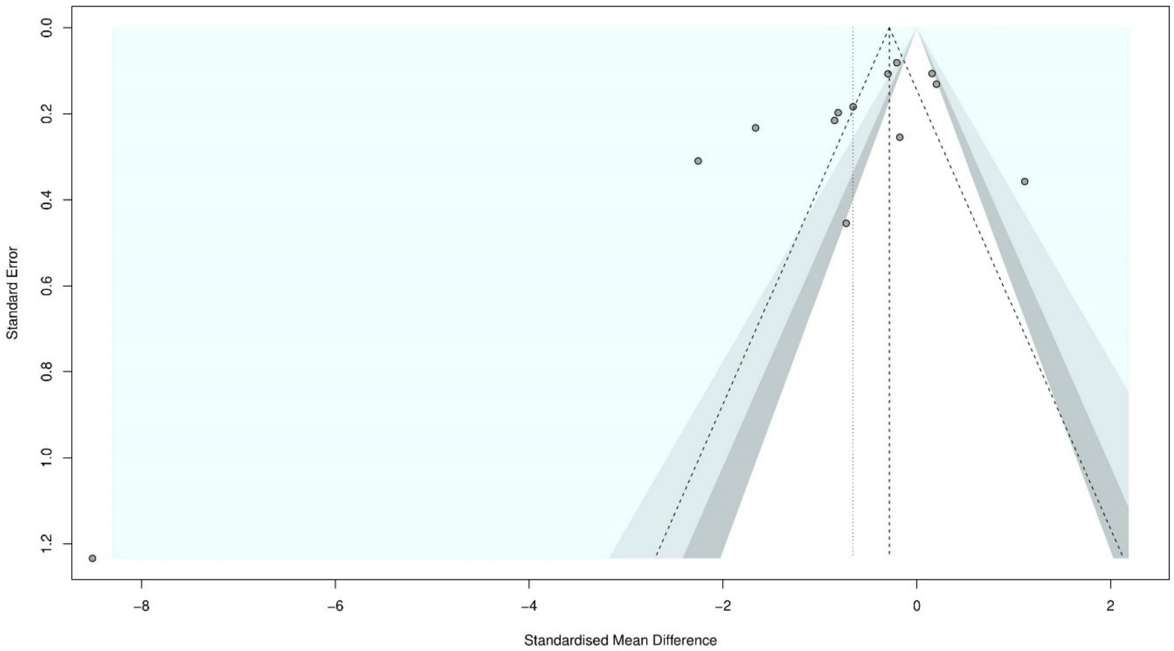

Figure S3: Funnel plot of the reporting bias.

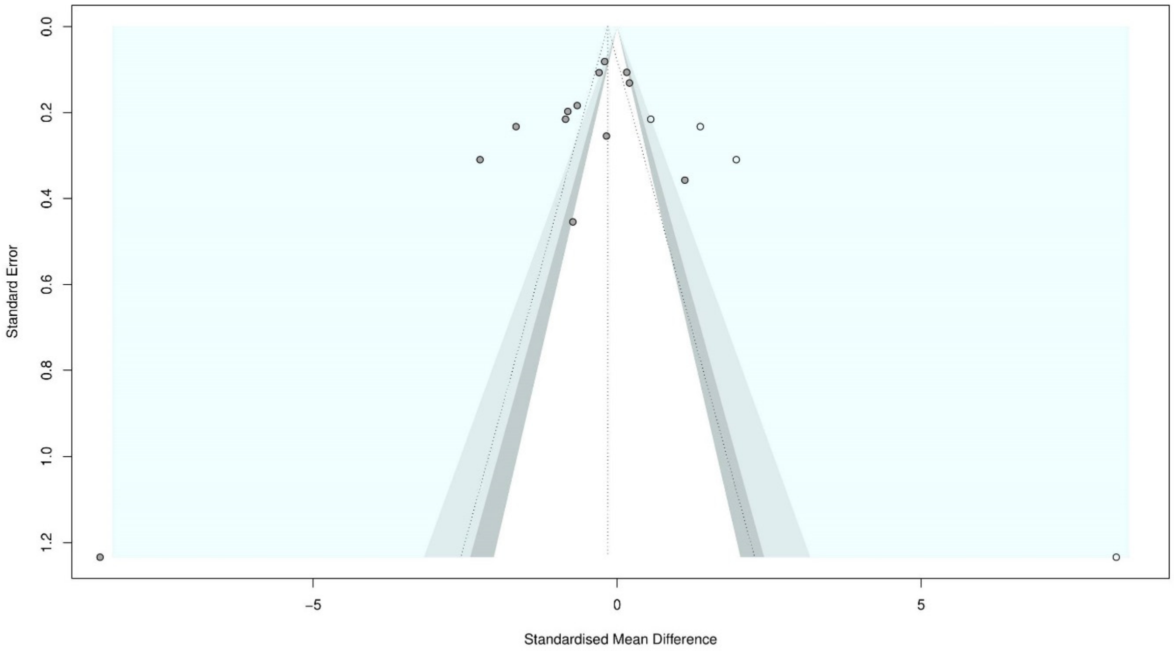

Figure S4: Trim-and-fill graph.
